# Immediate Epileptogenesis after Kainate-Induced Status Epilepticus in C57BL/6J Mice: Evidence from Long Term Continuous Video-EEG Telemetry

**DOI:** 10.1371/journal.pone.0131705

**Published:** 2015-07-10

**Authors:** Sreekanth Puttachary, Shaunik Sharma, Karen Tse, Edward Beamer, Abby Sexton, Joseph Crutison, Thimmasettappa Thippeswamy

**Affiliations:** 1 Department of Biomedical Sciences, College of Veterinary Medicine, Iowa State University, Ames IA 50011–1250, United States of America; 2 Institute of Aging and Chronic Disease, University of Liverpool, Liverpool, L69 3GA, United Kingdom; University of Modena and Reggio Emilia, ITALY

## Abstract

The C57BL/6J mouse as a model of seizure/epilepsy is challenging due to high mortality and huge variability in response to kainate. We have recently demonstrated that repeated administration of a low dose of kainate by intraperitoneal route can induce severe status epilepticus (SE) with 94% survival rate. In the present study, based on continuous video-EEG recording for 4-18 weeks from epidurally implanted electrodes on the cortex, we demonstrate that this method also induces immediate epileptogenesis (<1-5 days post-SE). This finding was based on identification of two types of spontaneous recurrent seizures; behavioral convulsive seizures (CS) and electrographic nonconvulsive seizures (NCS). The identification of the spontaneous CS, stage 3-5 types, was based on the behaviors (video) that were associated with the EEG characteristics (stage 3-5 epileptiform spikes), the power spectrum, and the activity counts. The electrographic NCS identification was based on the stage 1-2 epileptiform spike clusters on the EEG and their associated power spectrum. Severe SE induced immediate epileptogenesis in all the mice. The maximum numbers of spontaneous CS were observed during the first 4-6 weeks of the SE and they decreased thereafter. Mild SE also induced immediate epileptogenesis in some mice but the CS were less frequent. In both the severe and the mild SE groups, the spontaneous electrographic NCS persisted throughout the 18 weeks observation period, and therefore this could serve as a chronic model for complex seizures. However, unlike rat kainate models, the C57BL/6J mouse kainate model is a unique regressive CS model of epilepsy. Further studies are required to understand the mechanism of recovery from spontaneous CS in this model, which could reveal novel therapeutic targets for epilepsy.

## Introduction

Temporal lobe epilepsy (TLE) is the most common form of human epilepsy [1]. To understand the pathogenesis of human TLE, rodent models of epilepsy have been developed and characterized. A variety of methods have been tried in rodent models to induce status epilepticus (SE) and epileptogenesis [2–4]. Hitherto, rat models (for example [5–8]) and some mouse models (for example [9, 10]) of TLE are well characterized and extensively studied. Several weeks after the induction of SE in these models, intermittent or continuous video-EEG recordings have provided convincing evidence for the onset of spontaneous recurrent seizures, SRS (for example [11–13]).

Kainate is one of the most common drug used to induce SE in rodents (reviewed by [14–18]). Although several mouse models of epileptogenesis have been developed [19, 20], the C57BL/6J strain posed several challenges such as high mortality, inconsistent seizure response and resistance to kainate-induced neurotoxicity by the intraperitoneal (i.p.) route at a dose <30 mg/kg [21–24]. In the previous studies, a single dose of kainate by the i.p. route in these mice had failed to produce consistent severe SE without high mortality [25, 26]. Recently, we have addressed these issues in the C57BL/6J mice by administering kainate in repeated low doses (5 mg/kg i.p., at 30 min intervals), until they reach the stage-5 seizures [27].

In order to overcome the kainate-resistance to neurotoxicity in the C57BL/6J strain, by the i.p. or the subcutaneous (s.c.) route, kainate administration via the intra-hippocampal (for example [28, 29]), the intra-striatal [30] and the intra-amygdalar (for example [31]) routes were employed. It was known that severity of SE is the most important factor to induce epileptogenesis [32, 33]. Since the repeated low dose of kainate administration via the i.p. route could induce severe SE in the C57BL/6J mice with least mortality [27], we hypothesized that this approach could also induce epileptogenesis. To test this hypothesis, we induced mild to severe SE with kainate by intraperitoneal route in the C57BL/6J mice using the same method as described in our recent publication [27]. In the present study, after the induction of SE, the mice were subjected to continuous video-EEG monitoring for 4–18 weeks. Like in other models of epilepsy (for example [32, 33]), the frequency of spontaneous convulsive seizures (CS) directly correlated with the severity of the SE in the C57BL/6J mice. In addition, we present unexpected and interesting findings from this study: i) immediate epileptogenesis occurred in both the severe and the mild SE groups ii) the frequency of spontaneous CS increased with the severity of the SE but they decreased after 4–6 weeks iii) irrespective of the severity of the SE, the spontaneous electrographic nonconvulsive seizures (NCS) persisted throughout the observation period. These findings are presented and discussed in this study.

## Materials and Methods

### Animal source and ethical approval

The experiments were performed using the C57BL/6J male mice of 6–7 weeks old. They were purchased from the Jackson Laboratory, ME, USA and maintained in the Laboratory of Animal Resources at Iowa State University (ISU). The mice were housed under controlled environmental conditions (19°C– 23°C, 12 hour light: 12 hour dark), with *ad libitum* access to food and water. All experiments were performed according to the approved protocol by the Institutional Animal Care and Use Committee, ISU, USA (protocol no. 10-12-7446-MR). All surgeries were performed under isoflurane anesthesia and all efforts were made to minimize discomfort throughout the duration of the experimentation. All mice were euthanized with an overdose of pentobarbital sodium (100 mg/kg, i.p.) at the end of the experiments.

#### Terminologies

Duration of the entire SE, established SE, criteria for mild and severe SE classification, and the latent period.

**Fig 1:**
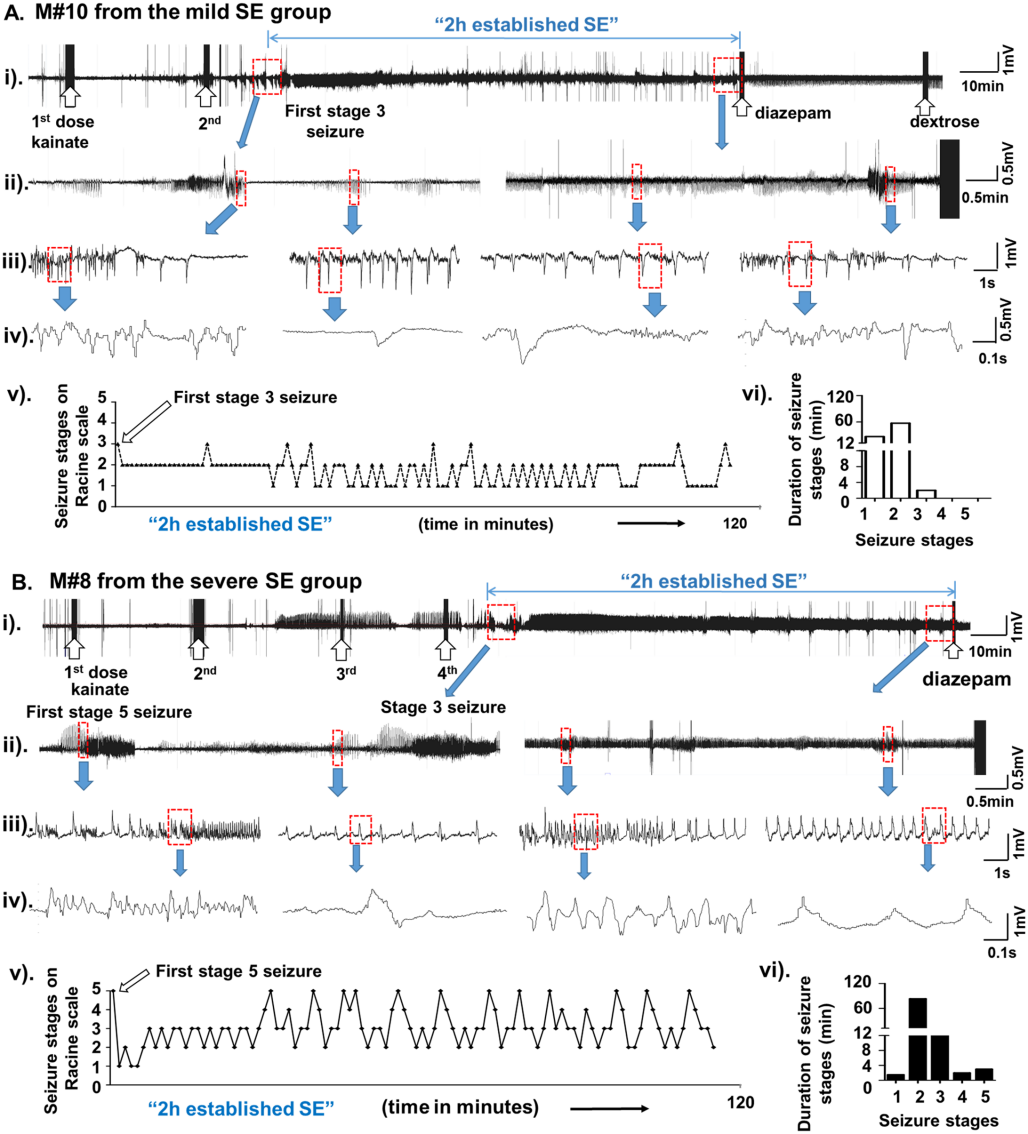
A (i to iv) and B (i to iv) are the representative EEG traces obtained during mild and severe SE, respectively, in this study. The behavioral scoring (Racine scale stages from 1 to 5) for these mice during the 2 hours established SE (v) and the duration of each stage was quantified (vi). The upward open arrows indicate the injections given [kainate (5 mg/kg)- first two arrows in A (i), and first four arrows in B (i); diazepam and dextrose at the other end of the trace]. The first episode of stage-3 seizure in the mild SE group (A, after the second dose of kainate) or the stage 5 in the severe SE group (B, after the fourth dose of kainate) is shown on the EEG traces (i and ii). The expanded EEG trace from the very end of the established SE, prior to the diazepam treatment, is shown in the panel (ii). A further expanded EEG traces are also shown in panels (iii) and (iv). The first stage 3 (in mild group) or the stage 5 (in the severe group) marked the beginning of the 2 hours established SE. During the 2 hour period, the mouse [panel A, (v)] had stage 2 seizures continuously for first 30 minutes with a second episode of stage-3 seizure half-way through and changed to the stage-1 seizure. Beyond this point, the remaining 90 minutes, the mouse (panel A) had continuous seizures ranging from stage-1 to stage-3. The exact amount of time spent at each stage is given in the panel (vi), likewise for the mouse in the severe SE group (panel B). During the 2 hour period, the mouse (panel B) had a brief stage-1 seizures followed by continuous stage 2–5 seizures during the remaining period of 2 hours (v). The exact amount of time the mouse spent at each stage is given in the panel vi.

Depending on the number of injections of kainate given, the overall duration of the entire SE varied from 3–6 hours. During this period of SE, all the mice had continuous epileptiform activity on EEG and the mice experienced continuous stage-1 and -2 (Racine scale 1 and 2) type behavioral seizures for greater than 10 minutes prior to the onset of stage 3 or 5 seizures (Racine scale 3 or 5). Once the mice reached stage 3 or 5 seizures, the behavioral and electrographic seizures fluctuated between stage 1 to stage-3 or -5 (Racine scale 3 or 5) for greater than 10 minutes. Overall, the SE in the present study met the criteria set by the International League Against Epilepsy [34, 35] i.e., SE was a continuous seizure activity that lasted for more than 10 minutes and also the different types of seizures (stage 1–5) were recurring at very short intervals (< 1 minute) during the established SE (Fig 1).

The “established SE” in the present study is the duration between the first onset of stage-3 or stage-5 seizure during the SE and the diazepam treatment, which is typically 2 hours (Fig 1, and also Illustrated by Tse et al., [27]).

To classify mice under the “mild SE”, the first stage-3 seizure and for the “severe SE”, the first stage-5 seizure were considered as the starting points for the 2hour established SE. The end point for both groups was when the diazepam was administered. During the 2 hours established SE, in the mild group the seizures fluctuated between the stage-1 to stage-3, while in the severe group between stage-1 (most often stage-2) to stage-5. The other criterion considered was that the mice under “severe SE” group should have had experienced a minimum of continuous 10 minutes of stage 3–5 seizures in addition to continuous stage 1–2 seizures for a long period (often > 40 minutes). The “mild SE” group mice should have had experienced stage-3 seizures intermittently for less than 10 min but continuous stage-1 or -2 seizures often greater than 40 minutes (for example, Fig 1).

The latent period is the period between the diazepam treatment and the first occurrence of spontaneous behavioral CS (“motor seizure latent period”) or the first onset of spontaneous electrographic NCS (“electrographic seizure latent period”). The period beyond the first spontaneous electrographic NCS was considered as the epileptic phase.

The “activity counts”, in the present study, refers to the counts that were generated due to locomotor activity. These behaviors were detected by the radiotransmitter and relayed to the computer as “activity counts/minute”. Higher the counts, greater the locomotor activity. The increased activity counts generated due to behavioral CS were associated with high amplitude and high frequency epileptiform spikes on the EEG and increased gamma power. The distinguishing feature of activity counts associated with normal behavior was the lack of EEG power in the gamma band. The activity counts due to exploratory or grooming behavior lacked high frequency and high amplitude epileptiform spike clusters on EEG. Further details on artifact spikes and epileptiform spikes, and activity counts in C57BL/6J mouse using epidural electrode technique has been published recently [27].

### Surgery, telemetry device implantation, SE induction with kainate and video-EEG monitoring

Twenty three mice were implanted with the telemetry device (Physiotel Multiplus ETA-F20, Data Science International, MN, USA), subcutaneously, 10 days prior to the induction of SE with kainate. The two electrodes were inserted into the burr holes bilaterally, 2.5 mm caudal to the bregma and 2.0 mm lateral to the midline, to record EEG from each hemisphere. The electrodes were positioned in contact with the dura mater over the surface of the cortex. The detailed surgical procedure for implanting the electrodes and the radiotransmitter has been described in our previous publications [27, 36].

The mouse cages were randomly allotted to the PhysioTel RPC receiver pads that transmit the data from the telemetry devices to Windows PC via the data exchange matrix. We used the Dataquest ART software to acquire real-time data at a sampling frequency of 1000 Hertz (Hz) and the NeuroScore software (DSI, MN, USA) to analyze the EEG recordings. The data acquired from the telemetry device included the EEG, the activity counts/minute and the mouse body temperature. The video-EEG recording was started soon after the surgery to acquire baseline EEG from each mouse that consisted of day and night cycles. The video was recorded at 25 frames/sec.

Nineteen out of 23 mice received kainate (Abcam, USA), which was prepared fresh in sterile distilled water at a concentration of 2 mg/ml. The remaining four mice received equal volumes of sterile distilled water, instead of kainate. These mice served as control. A repeated low dose of kainate at 5 mg/kg per injection was given i.p. at 30 minutes intervals. This method was useful to titrate the mice to achieve either mild or severe SE. The stages in the SE were identified and recorded according to the modified Racine scale (stage-1 to -5) [27]. Nine mice were titrated to achieve severe SE with a duration of >10 minutes of continuous stage 3–5 seizures during the 2 hours established SE. The 2 hour duration of the established SE started from the first onset of the stage-5 seizure to the time point when the mice were administered with diazepam (10 mg/kg, i.p.). The remaining 10 mice were titrated to achieve mild SE for a duration of <10 minutes of intermittent stage-3 seizures during the 2 hours established SE. In both mild and severe groups, all mice experienced continuous stage1-2 seizures for greater than 10 minutes. During the induction of SE, the behavior of the mice was video-EEG recorded and, simultaneously, two personnel directly scored the behavioral seizures based on the modified Racine scale [37], in 5 minutes epochs [27, 36]. The behavioral seizures during the SE were also further scored from the “standalone videos” by two other personnel who were blind to the experiments. The average score was used to calculate cumulative seizure severity score (CSSS) for the behavioral seizures.

The behavioral SE was terminated with diazepam, and immediately all the mice received dextrose normal saline (1 ml, s.c). From our experience, we knew that the mice with severe SE will lose their bodyweight (about 5%) in the first 2-3d of post-SE. To overcome this, dextrose normal saline injection were continued once a day and soft food pellets were provided until the mice regained their body weight (usually by third day). After the diazepam administration, the video-EEG recording was continued for 4–18 weeks.

The four (out of 23) mice that received sterile distilled water instead of kainate were also treated with diazepam and dextrose normal saline. Four injections of distilled water were given at 30 min intervals to match with the vast majority of the mice that received kainate in multiple injections. The diazepam was given 2 hours after the last dose of distilled water. These mice were also video-EEG monitored continuously for 4 weeks to investigate whether the surgery-induced trauma, and the implanted electrodes, also induce epileptogenesis or spontaneous spike-wave discharges or epileptiform spiking as reported for the rat models [38–40].

### CSSS index calculation to determine severity of the SE—based on the behavioral and the EEG characteristics during the SE

Four research assistants, who were unaware of the treatment groups, analyzed the video-EEG for severity of the SE and determined behavioral and electrographic CSSS indices. The details of the behavioral seizures at different stages, on Racine scale, during the SE in the C57BL/6J, induced by kainate, have been published [27, 36]. The duration between the first onset of the stage-5 seizure (severe SE group) or the stage-3 seizure (mild SE group) and the diazepam treatment was 2 hours. It is important to note that during the 2 hours of established SE, the seizures recurred continuously between stage-1 and stage-3 or -5. Behavioral CSSS (in minutes) for each mouse was calculated based on the exact amount of time the mouse spent at each stage of a convulsive seizure between stage-3 and stage-5 (total of all three stages from 3 to 5 in minutes for the severe group, stage-3 only in the mild group) during the 2 hour period. It is also important to note that the duration of continuous stage-1 and -2 seizures, which was often greater than 40 min in both severe and mild groups, was not considered to calculate CSSS index. The electrographic CSSS was calculated for both the groups based on the exact duration of stage≥3 epileptiform spikes on the EEG (total of all three stages from 3 to 5 in minutes for the severe group, stage-3 only in the mild group). The EEG characteristics were always correlated with the behavioral seizures (but certain behaviors during an episode were not always associated with the epileptiform spiking during the SE), spectral density characteristics and the activity counts [27]. To determine the SE as mild or severe, both the behavioral and the electrographic CSSS indices were considered.

### Identification and quantification of spontaneous CS based on the real-time integrated video-EEG-power band characteristics and the activity counts

We have previously described the procedure for EEG quantification during kainate-induced SE in the C57BL/6J mice [27]. In our previous short term study, a minimum of 24 hours baseline EEG was recorded from each mouse before administering the kainate. In the present long term study, we considered 10 days of continuous baseline recording which included day and night cycle, sleep and awake state, and resting and exploratory activities. The video-EEG recording was continued during and after the kainate treatment for up to 4–18 weeks. All the post-kainate responses were normalized against the baseline from the same mouse [8, 9, 27, 36]. The EEG raw signal in 10 seconds epochs, after manually excluding the artifacts, was subjected to Fast Fourier Transformation (FFT) to derive power bands (power spectral density). The EEG signal component containing various frequencies were split into individual power bands corresponding to the delta (δ, 0.5–4 Hz), the theta (θ, 4–8 Hz), the alpha (α, 8–12 Hz), the sigma (Σ, 12–16 Hz), the beta (β, 16–24 Hz) and the gamma (γ, 24–80 Hz) [27, 36]. The power in different spectra changed depending on the stage of a seizure within an episode and the power in some bands increased as a seizure progressed from stage-1 to -5. The baseline power for all the spectral bands were <5 mV^2^. To differentiate the epileptiform spikes from the normal baseline spikes or from the spikes due to electrical or mechanical artefacts, we considered individual spike characteristics such as amplitude, duration, frequency, inter-spike intervals and the activity counts (per minute) during this period. The spike amplitude threshold for the baseline was 100μV. The epileptiform spikes were detected using the NeuroScore software by setting the following parameters; amplitude threshold between 150 and 1500μV with the individual spike duration 15 and 500ms, inter-spike interval 80 and 5000ms, and the spike train join interval was 100 and 5000ms within an episode (Table 1). After detecting seizure episodes using the NeuroScore software, the EEG recordings were manually verified and all the recurrent spontaneous CS types were checked against the real-time videos, the spectral bands and the activity counts. The artifacts such as exploratory behavior and electrical interference were detected based on the individual spike characteristics, the power spectrum, and the activity counts [27].

**Table 1:** The epileptiform spike characteristics for the spontaneous behavioral CS and the electrographic NCS and during the post-SE period in the C57BL/6J mice. The spikes/min column represents a minimum and a maximum number of spikes/min in an episode. The detailed explanation for the epileptiform spikes on the EEG that were associated with the behavior during the SE, and artifacts, are given elsewhere [27].

**Seizure stages**	**Length of episode (sec)**	**Spike Amplitude (μV)**	**Inter-spike interval (ms)**	**Spike frequency/ min(within an episode)**
**Stage-1 inter-ictal spikes**	300–1500	200–300	1000–4000	15–60
**Stage-2 inter-ictal spikes**	300–1500	400–1500	2000–5000	12–30
**Stage-1 episode**	10–15	200–300	200–500	120–300
**Stage-2 episode**	20–40	400–1500	800–1200	48–72
**Stage-3 episode**	20–50	500–1500	100–300	180–600
**Stage-4 episode**	30–60	500–1500	100–300	180–600
**Stage-5 episode**	40–120	500–3000	80–300	180–720

### Identification and quantification of spontaneous electrographic NCS based on the EEG and the power band characteristics

The spontaneous electrographic NCS episodes included the stage-1 and -2 type epileptiform spike clusters along with their respective pre-, post- and inter-ictal spikes. The stage-1 electrographic NCS episode included the stage-1 epileptiform spikes along with its associated stage-1 pre- and post-ictal spikes. Similarly, the stage-2 electrographic NCS episode included the stage-2 epileptiform spikes with its associated stage-2 pre- and post-ictal spikes. The duration of some of the spontaneous electrographic NCS episodes, usually the stage-2 type, were as long as 30 minutes with an inter-spike interval of 2-5s (frequency 0.2–0.4 Hz, Table 1), but we set a minimum of 12 seconds duration with a frequency ≥0.8 Hz to consider it as an electrographic NCS episode.

The numbers of spontaneous CS (stage -3,-4 and -5) and the spontaneous electrographic NCS episodes (stage-1 and -2) were plotted as group data for the duration of first 4 weeks, 5–8 weeks, 9–12 weeks and 13–18 weeks for both the severe and the mild SE groups. The spontaneous CS occurred during the first 4 weeks after the SE, in both the severe and the mild SE groups, are also presented at 24 hour epochs to demonstrate mouse-to-mouse variability. The Mann-Whitney test was used to compare between the behavioral and the electrographic CSSS, and to compare between the total numbers of spontaneous seizures at different time points. The Kruskal-Wallis test was used to compare the seizures within the groups across different time points.

## Results

### The CSSS index to determine severity of the SE

As previously noted, there was no correlation between the CSSS index and the amount of kainate administered to achieve the SE [27]. All the mice in this study had continuous behavioral and electrographic stage 1–2 seizures for a minimum of 10 minutes prior to the onset of first stage-3 or -5 seizures and during the 2 hours established SE. After the onset of stage-3 or -5 behavioral seizures, the vast majority of the mice had continuous seizures that fluctuated between the stage-1 and the stage-3 in the mild group and between the stage-1 or 2 and the stage-5 in the severe group (for example, Fig 1). The severe SE group mice always had the stage-5 behavioral seizures that were characterized by generalized tonic clonic convulsions with lateral recumbence or jumping and/or wild running followed by generalized convulsions. The mild SE group mice always had the stage-3 behavioral seizures consisting of rearing with facial automatisms and forelimb clonus but never experienced generalized convulsions i.e. the stage-5 seizure. The video demonstrating the stage-3 to -5 in the C57BL/6J mouse has been published by our group [27]. In the vast majority of the mice, in both groups, the stage-1 and -2 seizures persisted continuously for > 40 minutes (for example, Fig 1). The representative EEG trace and behavioral seizures score, during the 2 hour established SE, from the mouse that had severe or mild SE is demonstrated in Fig 1.

**Fig 2:**
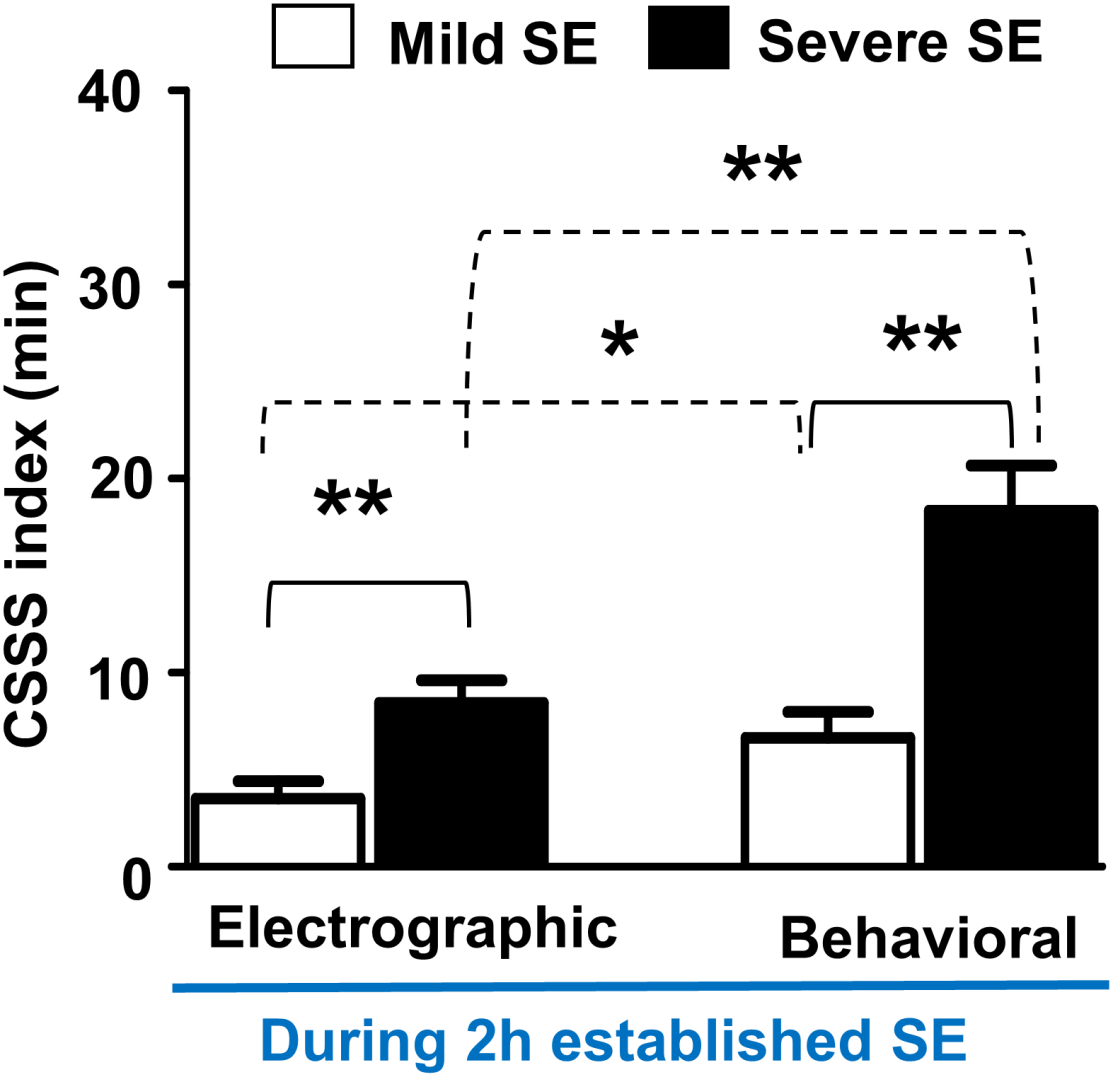
Behavioral and electrographic CSSS indices comparison between the severe and the mild SE groups during the 2 hours established SE. The CSSS indices for both behavioral and electrographic seizures were significantly higher in the severe SE group, when compared to the mild SE group (**p = 0.0047, electrographic CSSS; **p = 0.0011, behavioral CSSS; n = 9, Mann-Whitney test). There was almost 50% difference between the electrographic and the behavioral CSSS indices in both groups (*p = 0.037 for the mild group, **p = 0.0023 for the severe group, Mann-Whitney test).

The severe SE group mice that experienced stage-3 to -5 behavioral seizures had an average of 20 behavioral CSSS index and 10 electrographic CSSS index, while the mild SE group mice had only the stage-3 behavioral seizures intermittently for a period <10 minutes, and their CSSS indices were well below the severe SE group (Fig 2). The CSSS indices for both the behavioral stage ≥3 seizures and their associated EEG seizures were significantly higher in the severe SE group when compared to the mild SE group (p = 0.0047 for the electrographic CSSS and p = 0.0011 for the behavioral CSSS, the Mann-Whitney test; n = 9 each). Interestingly, there was about 50% difference between the behavioral and the electrographic CSSS values in both groups (Fig 2).

### Both severe and mild SE induce immediate epileptogenesis

**Fig 3:**
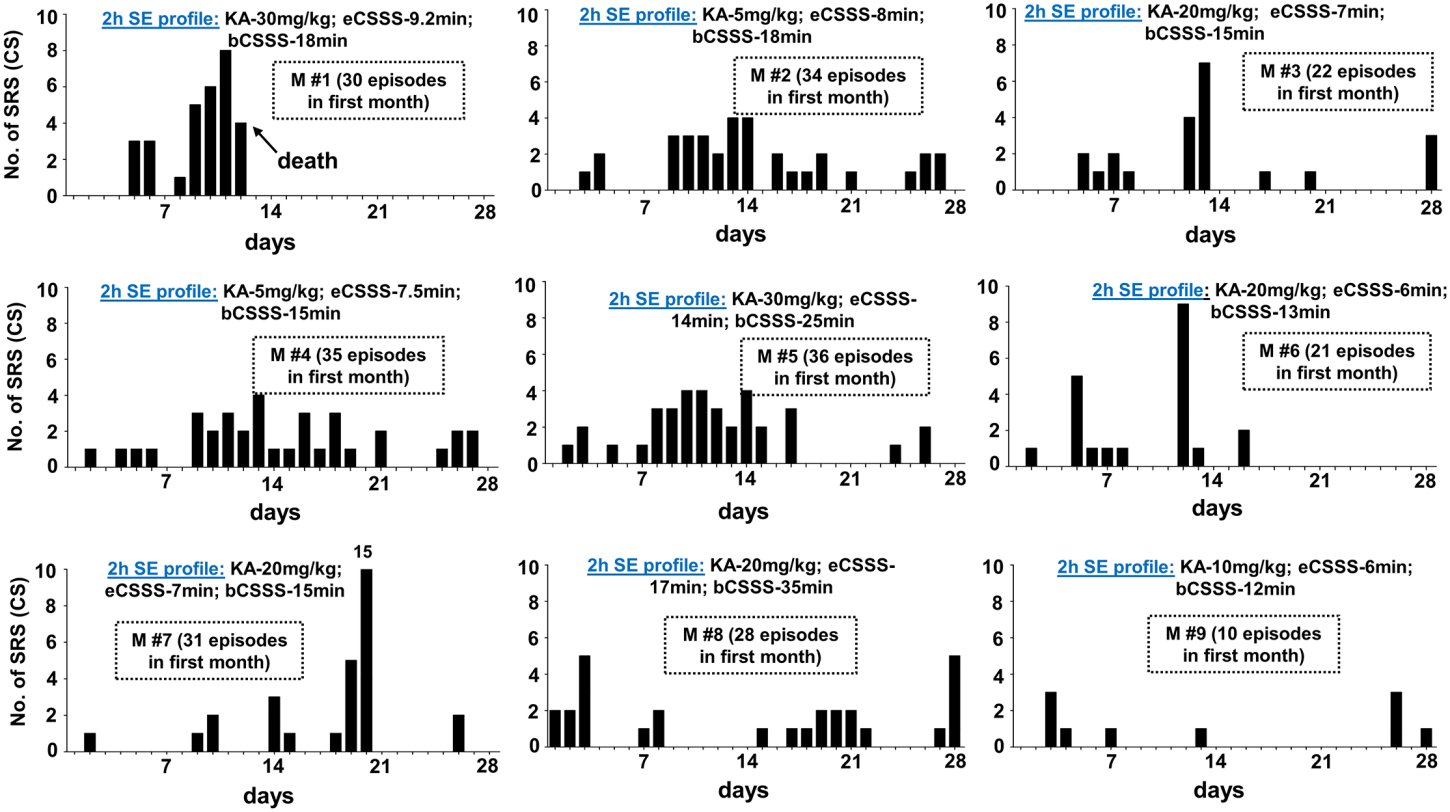
The frequency of spontaneous behavioral CS occurrence during the first 4 weeks of post-SE in the severe SE group. The 2 hour established SE profile, and the total numbers of spontaneous CS episodes/day during the first 4 weeks of post-SE are given for each mouse. All mice in the severe group had continuous stage-1 or 2 seizures for >40 min during the 2 hours established SE. There is no correlation between the amount of kainate received and the motor seizure latent period, and the numbers of spontaneous CS episodes. eCSSS = electrographic CSSS index (in min); bCSSS = behavioral CSSS index (in min).

**Fig 4:**
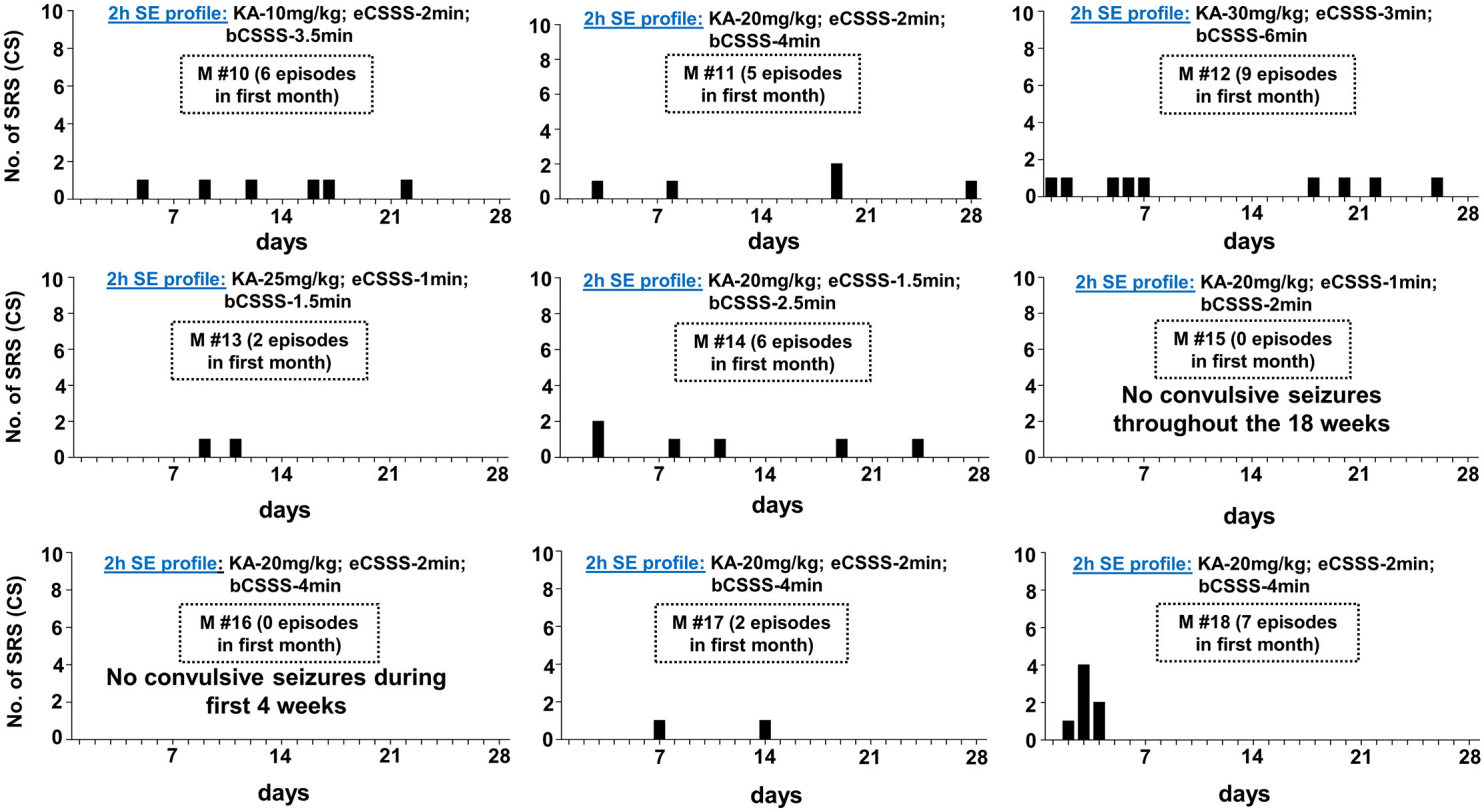
The frequency of spontaneous behavioral CS occurrence during the first 4 weeks of post-SE in the mild SE group. The 2 hour established SE profile, and the total numbers of spontaneous behavioral CS are given for each mouse. All mice in the mild group also had continuous stage-1 or 2 seizures for > 40 min during the 2 hours established SE (an example is shown in Fig 1). As in the severe SE group, there was no correlation between the amount of kainate given and the motor seizure latent period, and the number of CS episodes. Two mice did not become epileptic during first 4 weeks. eCSSS = electrographic CSSS index (in min); bCSSS = behavioral CSSS index (in min).

In this study, the period between the diazepam treatment and the first onset of spontaneous behavioral CS was considered as the “motor seizure latent period” while, the first onset of electrographic NCS was considered as the “electrographic seizure latent period” [8]. Interestingly, as in the rat kainate models of epileptogenesis/epilepsy described in the literature [8, 12, 17, 41], there was no well-defined prolonged ‘motor seizure latent period’ for the C57BL6/J mice, in both groups, in this study. The first electrographic NCS was observed at 138 ± 66 minutes in the mild group and at 78± 60 minutes in the severe group after the diazepam treatment. There was no significant difference between the groups. The motor seizure latent periods for the mild and severe groups were 7±3.8 (n = 8, one being an outlier, 33 days) and 1.8±0.47 days (n = 9, mean ±SEM), respectively, and there was also no significant difference between the groups. Out of 9 epileptic mice under the severe SE group, one mouse had no motor seizure latent period, 4 mice had 1d, and 2 mice each had 2 and 4 days (Fig 3). In the mild SE group, out of 10 mice one mouse showed neither a behavioral CS nor an electrographic NCS throughout the 18 weeks period. Another mouse (Fig 4, M#15) in this group had only the electrographic NCS throughout the 18 weeks but no behavioral CS were observed. The motor seizure latent period in the mild SE group varied from 1 to 10 days (Fig 4) with an exception of 33 days in one mouse (M#16). In both mild and severe SE groups, spontaneous electrographic NCS always occurred prior to the behavioral CS.

**Fig 5:**
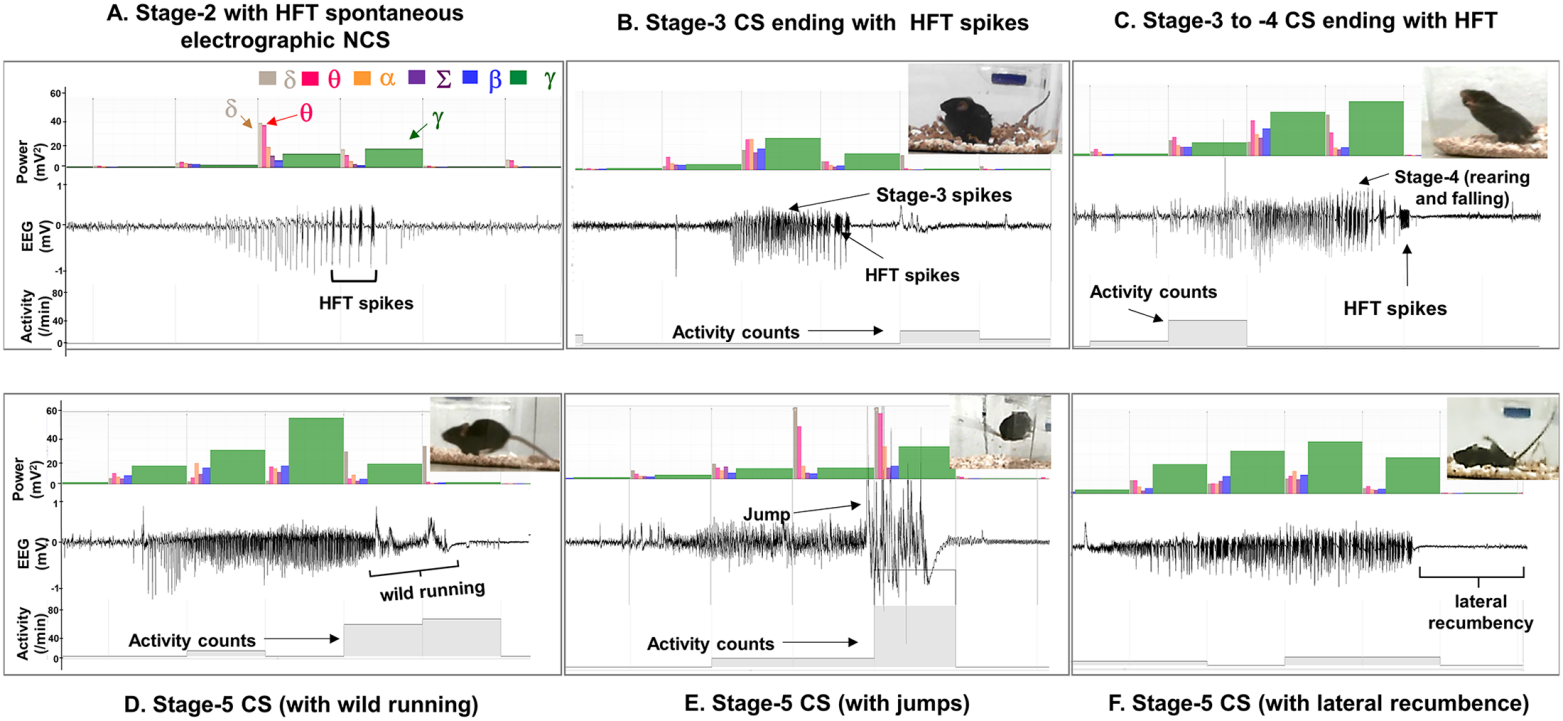
Types of spontaneous electrographic NCS (A) and behavioral CS episodes (B-F) observed during the first 4 weeks. (A) The stage-2 type of spontaneous electrographic NCS ending with the HFT spikes had no behavioral correlates. Five types of spontaneous behavioral CS were identified. (B) the stage-3 type ending with the HFT spikes (C) the stage-3 to -4 type episode ending with the HFT spikes, (D-F) the stage-5 episodes preceded by the stage-3 and -4 type spikes with wild running (D) or without wild running and/or ending with lateral recumbence/rigidity (F), which is characterized by low amplitude spikes on the EEG compared to the baseline. (E) The stage-5 episode was preceded by the stage-3 and -4 types followed by several jumps and may end with lateral recumbence/rigidity. In all the spontaneous CS types, the stage-3 component increases the gamma power while, the increase in the activity counts corresponds to the movements due to seizures during this period. The gamma power decreases in the stage-5 except during jumping and wild running, which also coincides with the increased activity counts.

All the spontaneous behavioral CS, evident from the real-time videos, were always associated with the EEG and the power spectral characteristics, and the activity counts (Fig 5B–5F). The mice that became epileptic had the stage-3 to -5 types of spontaneous behavioral CS (Fig 5B–5F) and/or the stage-1 to -2 types of spontaneous electrographic NCS (Fig 5A). A few video examples of spontaneous behavioral CS with their associated EEG characteristics, the power spectrum, and the activity counts can be found in the online videos (S1–S3 Videos). In all the 5 types of spontaneous behavioral CS episodes, identified in this study, there was an increase in the gamma, theta and delta powers. These were due to the stage-3 spikes and/or the stage-5 spikes that were associated with wild running or jumping behavior. These behaviors were detected by the radiotransmitter and displayed on the screen as the “activity counts/minute” that is shown at the bottom of each episode (Fig 5; and S1–S3 Videos). These were also correlated with the behavioral CS, as evident from the video, and increased the gamma power on the EEG. All of these parameters were used to confirm spontaneous behavioral CS episodes. However, the stage-4 behavioral CS (repeated rearing and falling) sometimes lacked activity counts when there was a limited movement (Fig 5C). The gamma power decreased during the stage-5 spikes when it was associated with the lateral recumbence or rigidity/restricted movement that coincided with the decreased activity counts (for example in Fig 5F). This pattern was also observed during the post-ictal depression (Fig 5B–5F). We could clearly distinguish the increased activity counts and the gamma power due to movement artifacts from the real seizure activity [27].

### The spontaneous behavioral CS episodes decreased after the 4 weeks of SE but the spontaneous electrographic NCS continued to exist during the 18 weeks observation period

**Fig 6:**
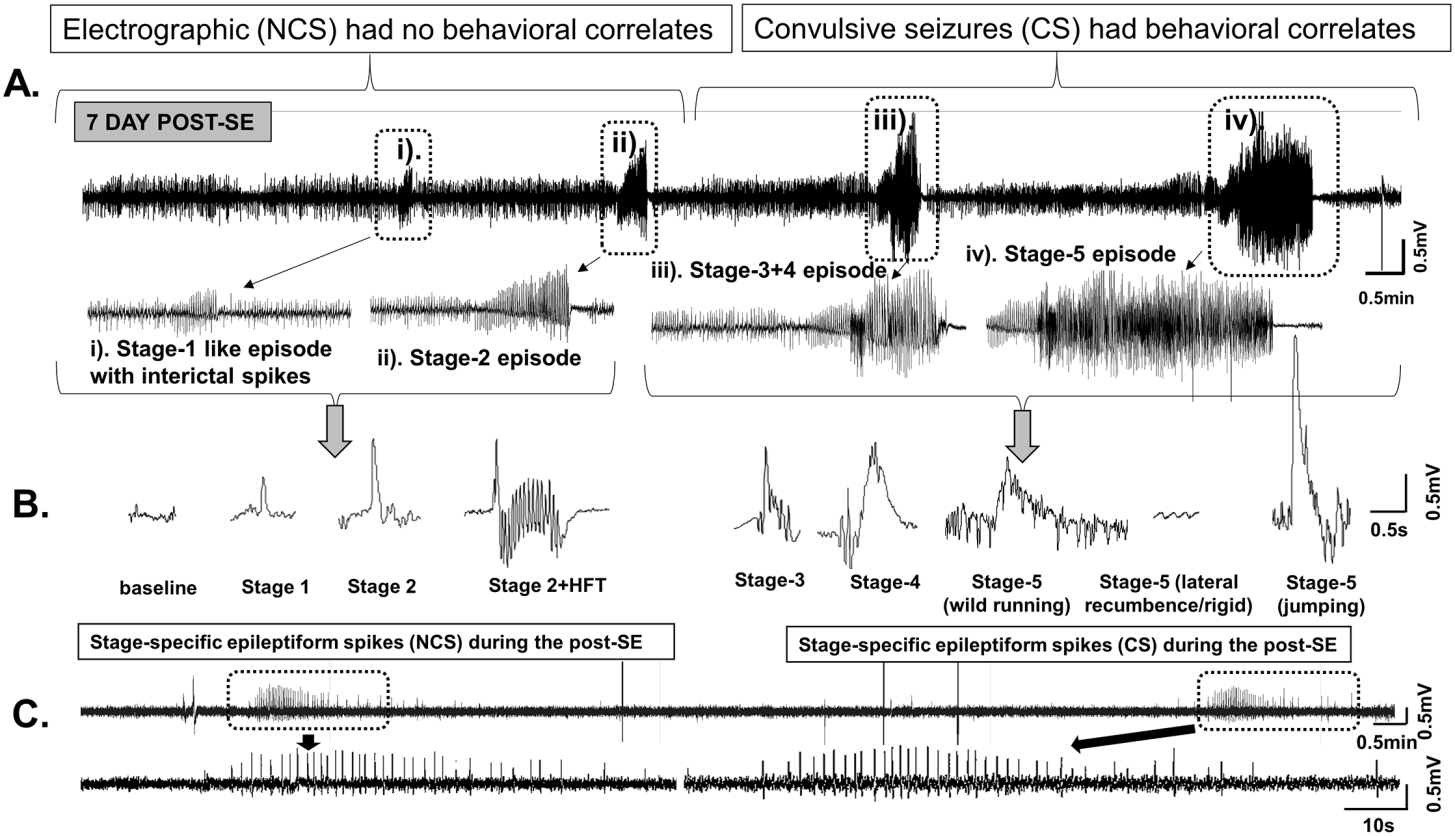
The EEG features of the spontaneous electrographic NCS and behavioral CS. A. A 20min EEG trace from the mouse at 7d post-SE. A pattern of the stage-1 and the stage-2 type NCS episodes (with no behavioral correlates unlike during the SE) preceded the stage 3–5 behavioral CS episodes. After 4 weeks, the stage 3–5 episodes were reduced, however the stage-1 and -2 continued to persist. B. The spike characteristics during the post-SE period. The HFT spikes reduced after the 4 weeks in all types of seizures in both the mild and severe SE groups. An example of an electrographic NCS on its own (without progressing to stage 3–5 seizure) is also shown in the panel C- a 20min trace showing spontaneous electrographic NCS of stage 2 type spiking at 32 day post-SE (from the severe SE group). Such electrographic NCS episodes persisted throughout the 18 weeks in the severe and the mild groups.

We quantified the spontaneous behavioral CS (stage 3–5) and the spontaneous electrographic NCS (stage 1–2) from both the severe and the mild SE groups. An example of an EEG trace from a mouse at 7d post-SE (Fig 6A) illustrates the stage 3–5 type spontaneous seizures that occurred in less than 3 minutes after the stage 1–2 electrographic NCS. This was the EEG pattern seen in all the epileptic mice in both groups during the first week. After the first week, the types of behavioral CS shown in the Fig 5 were most commonly observed in both groups. The EEG characteristics (Fig 6B) during the spontaneous behavioral CS were similar to the pattern observed during the SE. The spontaneous recurrent electrographic NCS were identified based on the stage-1 and stage-2 epileptiform spike characteristics on the EEG (Fig 6B) and increased power in the theta and delta bands, which were also similar to the EEG features identified during the SE in the present study and in earlier studies from C57BL/6J mouse kainate model [27, 36]. The spontaneous electrographic NCS, presented as stage-2 clusters, contained “high frequency trigger” (HFT) spikes during the first 4 weeks (an example is shown in Fig 5A). However, the HFT spikes were rarely found in stage-2 clusters during the 5–18 weeks post-SE (for example Fig 6C, the EEG trace from 32 day post-SE is shown). A random analyses of three weeks video-EEG recordings revealed that about 80% of the electrographic NCS were associated with behavioral phenotypes (observed from high resolution “standalone videos” rather than from the videos that were integrated with the EEG files). The remaining 20% of NCS episodes were the stage-2 with HFT spikes which lacked behavioral correlates. The HFT pattern observed in the C57BL/6J mouse has been described elsewhere [27].

**Fig 7:**
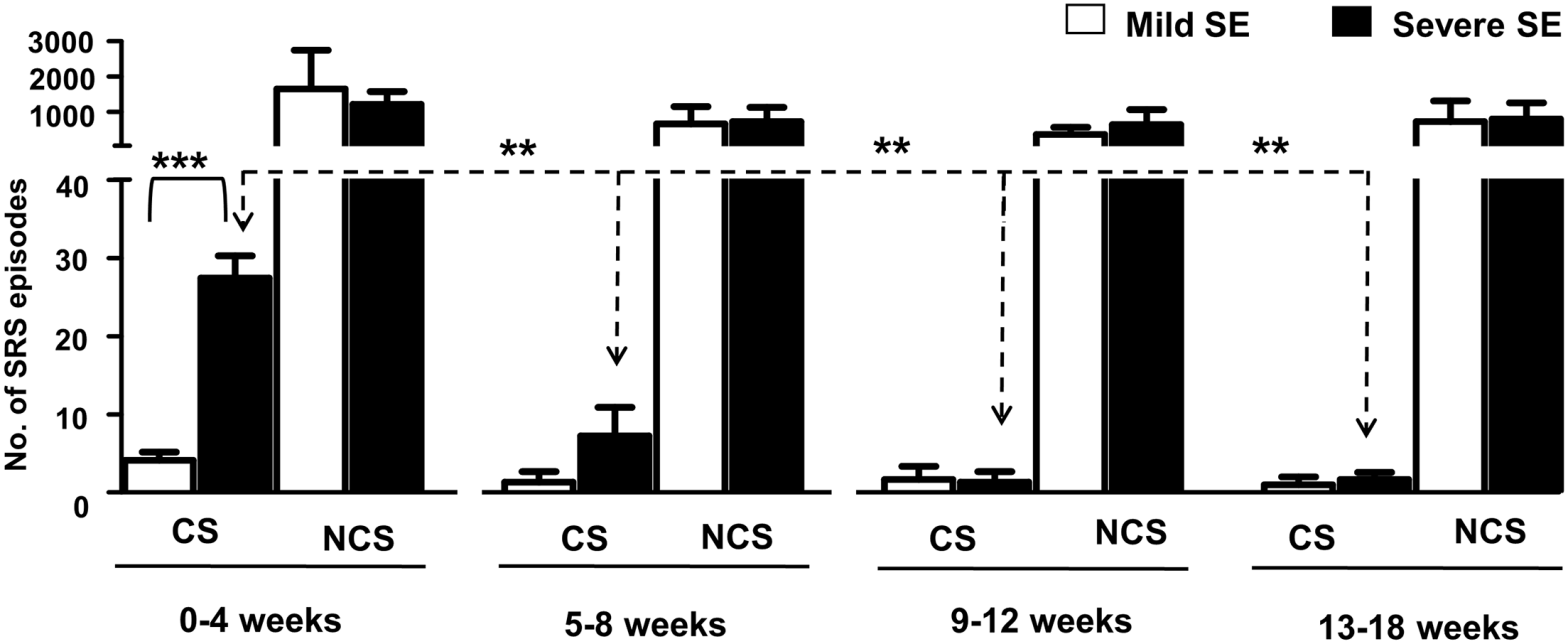
Comparison of the spontaneous behavioral CS and the spontaneous electrographic NCS occurrence in the severe and mild SE groups from 0–18 weeks. There was a significant increase in the number of behavioral CS episodes during the first 4 weeks in the severe SE group when compared to the mild SE group (***p = 0.0004, Mann-Whitney test, n = 9 each). The spontaneous CS in the severe group significantly reduced during 5–8 weeks or 9–12 weeks or 13–18 weeks when compared to 0–4 weeks (p = 0.0026, Kruskal-Wallis test). However, there was no significant difference in the spontaneous behavioral CS in the mild group across different time points. Further, spontaneous electrographic NCS episodes did not significantly change between the two groups at any time point and continued to exist throughout the 18 weeks period.

During the first 4 week period, all the mice in the severe SE group were epileptic and had 27±3 episodes of spontaneous behavioral CS (stage ≥ 3). In the mild SE group, except three mice the rest had 4±1 behavioral CS during this period (Fig 7; p = 0.0004, severe SE versus mild SE group, Mann-Whitney test). After 4 weeks, the number of spontaneous behavioral CS significantly decreased in the severe group (Fig 7; p = 0.0026, 0–4 week versus the rest of the time intervals for up to 18 weeks; Kruskal-Wallis test). And there were no significant differences in the numbers of spontaneous behavioral CS between the severe and the mild groups during the 5–18 weeks period (Fig 7). Interestingly, there were also no significant differences in the numbers of the spontaneous electrographic NCS episodes between the severe and the mild groups throughout the 18 week period, including the first four weeks of post-SE (Fig 7). During the 18 week post-SE period, the mice in both the severe and the mild SE groups had ~20 spontaneous electrographic NCS type episodes per day/night cycle.

None of the four control mice that were monitored continuously for 4 weeks or the baseline EEG recordings from the 19 mice for 10 days (before the induction of SE) from the experimental groups had any epileptiform spikes either in isolation or in clusters. None of the mice from the control or the severe SE or the mild SE group had infection at the site of the electrodes or at the site of the radiotransmitter. There was no evidence of damage to the brain by the implanted electrodes. The mice body temperature, detected by the radiotransmitters in real-time, was also constant throughout the course of the experiment.

## Discussion

The results of this study demonstrate that both severe and mild SE in C57BL/6J mice induce epileptogenesis soon after the SE. Severe SE increases the numbers of spontaneous behavioral CS during the first 4 weeks but they decrease thereafter. Mild SE also induces epileptogenesis but the numbers of spontaneous CS in the first 4 weeks were fewer than those found in the severe group. In both the severe and the mild SE groups, the spontaneous electrographic NCS continue to exist during the 18 weeks observation period. In both groups, all the spontaneous behavioral CS (stage 3–5) were associated with: characteristic EEG patterns (high frequency and high amplitude epileptiform spike clusters), the increased gamma, theta and delta powers in the power spectrum, and the increased activity counts. Considering the large numbers of spontaneous electrographic NCS occurred during the 18 weeks continuous study, a random observation of three weeks recordings revealed that about 80% of the electrographic NCS were associated with behavioral phenotypes in both groups. The remaining NCS episodes contained the stage-2 HFT pattern (for example, Fig 5A) which lacked behavioral correlates. The spontaneous electrographic NCS (EEG) patterns quantified were similar to the EEG patterns observed during the SE in the present study, and also to our previous studies from C57BL/6J mouse model [27, 36]. The NCS were also characterized by the increase in power in delta and theta bands.

### Severity of the SE, determined by the EEG and the behavioral characteristics, and its impact on latent periods, epileptogenesis and chronic epileptic period

The results from this study were based on continuous video-EEG recording from epidurally implanted electrodes on the surface of the cortex 10 days prior to the induction of SE. To our knowledge, there is little or no evidence of continuous (24/7) integrated video-EEG recording from C57BL/6J mice for a longer duration. Early epileptogenesis, less than a week after the SE, is also reported in the rat pilocarpine model [33, 42, 43, 44]. A similar observation was also made from the rat electroconvulsant model [7] and the pilocarpine mouse model of epilepsy [19]. These findings, except the study from Jung et al [44], were also based on continuous video-EEG monitoring, challenge the notion of “prolonged motor seizure latent period” after the induction of SE in rodent models.

In this study, there were no significant differences in the latency to the first onset of spontaneous electrographic NCS and behavioral CS between the mild and SE groups. And the NCS always occurred prior to the CS in both groups, which is in agreement with the other models of TLE [33, 43]. The effects of duration and severity of SE on latent period, epileptogenesis and chronic epileptic phase has been adequately addressed in rodent models of epilepsy [19, 32, 33, 43, 45]. It has been proposed that the duration of the latent period, whether for NCS or CS, depends on the duration of behavioral SE and the method of termination of SE in rodent models [43, 45]. In a pilocarpine rat model in which the behavioral seizures were terminated by diazepam and ketamine, no significant differences were found in latency to the onset of spontaneous NCS or CS between the rats that had 30 minutes or 120 minutes SE [33]. In another pilocarpine rat model study in which SE was terminated by administering high dose of diazepam (20 mg/kg), it has been reported that the longer duration of SE progressively delays spontaneous seizure onset [45]. However, in the pilocarpine mouse (the NMRI strain) [19] and the rat model used by Klitgaard et al [32] and Bortel et al [33], about 30 minutes of SE was sufficient to induce epileptogenesis. It must be emphasized that in the present study all the mice had established SE for 2 hours that included seizures of various stages ranging from stage 1 to stage 3 or 5 (for example, Fig 1). It is important to note that in the present study we have considered the exact duration of the behavioral CS stage ≥3 and their associated EEG seizure duration to calculate the severity index (CSSS indices) for each mouse. The behavioral CSSS index was 50% higher than the electrographic CSSS index for the SE in both groups suggesting that the behavioral CS, during the SE, originated from the brain are due to a widespread effects of kainate. Since only two epidural electrodes were used in this study, it would require a multi electrode system to record electrical activity from different parts of the brain to understand exaggerated locomotor behaviors caused by kainate during SE.

Interestingly, the behavioral SE was not terminated in some rat kainate models of TLE [3, 7, 17]. And in one such study, the first spontaneous electrographic NCS was recorded in less than 24 hours in 5 out of 9 rats [9]. In the present study, electrographic NCS occurred in both mild and severe groups in less than 2 hours after terminating the behavioral SE with diazepam. A recent study reveals that the domoic acid, an analogue of kainate, has a beta half-life of about 5 hours in rodents [46], which may suggest that very early NCS could be due to the residual effects of kainate in this study. However, the occurrence of first spontaneous CS in this study were unlikely due to the residual effects of kainate since they occurred during 24–36 hours after the last dose of kainate, the earliest time point observed in 2 out of 18 mice. The spontaneous CS observed in this study were also not due to surgery-induced trauma to the brain (the electrodes were placed epidurally on the surface of the cortex and the brains were always checked for electrodes-induced trauma after euthanasia) or infection or due to increased body temperature. In a rat kainate model of TLE, it has been reported that both hippocampal leads and the dural EEG lead show qualitatively similar activation throughout the recording period for all kainate-treated rats in that study [8]. This supports that the EEG signals originated from epidurally placed electrodes on the surface of the cortex in our experiments represents overall brain electrical activity during the SE and post-SE periods. Our previous studies from the C57BL/6J mouse kainate model using a similar technique, epidural electrodes on the surface of the cortex, demonstrated a correlation between the EEG changes and the changes occurring in the hippocampus bilaterally, for example increased Fos activation and reactive gliosis, and their modulation by an intervention drug during and after the SE [36].

The frequency of spontaneous behavioral CS was higher in the severe group than the mild group (both groups had received a similar range doses of kainate), implying that the severity of the SE influences the frequency of spontaneous CS. A similar findings were reported from the rat pilocarpine model by Bortel et al [33]. In contrast, the pilocarpine rat model study from Klitgaard et al [32], based on intermittent EEG recording, reported that the prolonged behavioral SE decreases the seizure frequency.

### The route of administration of kainate to induce epileptogenesis: intraperitoneal versus other routes

Inducing severe SE using kainate via the intraperitoneal route in C57BL/6J mice had been a challenge due to high mortality and inconsistency in their seizure response to a single dose of kainate [25, 26]. We have recently addressed these important issues by repeated administration of a low dose of kainate via the i.p. route [27]. The previous studies from systemic administration of kainate via the i.p. or the s.c. route in the C57BL/6J mouse have demonstrated resistance to kainate-induced neurotoxicity at a dose less than 30 mg/kg [25, 26, 47–50]. In order to overcome the resistance and to induce severe SE, in view of achieving epileptogenesis in C57BL/6J mice, focal injections of kainate by the intra-hippocampal [10, 28, 51–54] or the intra-amygdalar [55] route has been successfully attempted. A similar method of unilateral intra-hippocampal kainate injection in the guinea pig induced focal epilepsy [56–57]. The focal injection methods also induced early epileptogenesis by 3–5 days post-SE in mice [51–53, 55, 58], and 2–3 weeks to several months in the other studies [10, 28, 51–53, 55, 58]. Two studies [10, 55] have recorded EEG, but not video, continuously for 2–4 weeks, while the other studies recorded both video and EEG, but only intermittently [28, 51–53, 55, 58]. The choice of timings for intermittent recordings in these studies were based on the reported “motor seizure latent period of 2–3 weeks for the Swiss mice” [for example, 59]. The intranasal route for kainate administration has also been tried in the C57BL/6J mice to overcome the kainate resistance with limited success [60]. The focal injections of kainate although produce severe SE and spontaneous recurrent seizures, these techniques are labor intensive, technically demanding and expensive. Moreover, the focal administration of kainate is done under general anesthesia, which could add further variables to the experiments during the SE, and also the technique could damage the brain [61–63]. The i.p. route for administering kainate to induce SE is convenient and cost effective for non-telemetric studies. The systemic injection of kainate causes widespread neuronal loss in different parts of the brain in rats and in some mouse strains [64–67]. Since C57BL/6J mice are resistant to the direct effects of kainate—induced neurotoxicity, a further investigation is required to understand whether the severity of the SE, induced by kainate, cause a widespread neuronal loss, in these models, as reported for the rat kainate models (for example [3, 68]).

### Spontaneous behavioral CS decline after 4 weeks of the SE and the electrographic NCS continue to persist beyond 4 weeks

The most interesting and surprising result from this study is that the spontaneous behavioral CS decrease after 4 weeks of the SE. Unlike the rat models of kainate-induced acquired epilepsy and post-stroke epilepsy models [12, 69, 70] or rat pilocarpine models [32, 33] which are progressive, the C57BL/6J mouse kainate model is a regressive type. However, the spontaneous electrographic NCS continued to exist throughout the 18 weeks and their numbers did not change over time in both the severe and the mild SE groups, which suggest that these could serve as a chronic model for complex seizures. There were no differences in the numbers of electrographic NCS between the mild and severe SE groups at any time points in the 18 weeks continuous study (Fig 7). In contrast, in the rat pilocarpine model the NCS were 20% higher in severe SE compared to the mild SE rats in the 17-day continuous video-EEG study [33]. However, the frequency of convulsive seizures was higher in the severe SE rats in pilocarpine model, and they were progressive in both mild and severe SE groups [33]. In the present study, the behavioral CS frequency was also higher in the severe SE group when compared with the mild SE group but they decreased after 4 weeks in both groups (Fig 7). The reasons for the decrease/complete absence of spontaneous behavioral CS, in some mice, require further investigation. Identification of compensatory mechanism/s of recovery from behavioral epileptic phase could reveal a potential therapeutic targets for epilepsy. Our ongoing intensive video-EEG analysis from 4 weeks onwards in these mice revealed an important pattern in their EEG traces. The stage-2 type electrographic NCS during the late post-SE period (>4 weeks) more often lacked the HFT spikes, which were usually present during the first 4 weeks (for example, Fig 5A). We have recently described the importance of the HFT spikes in transition from stage-2 to stage-3, i.e., from nonconvulsive to convulsive seizure during the SE [27]. Perhaps, the absence or low numbers of HFT spikes on the EEG in some NCS clusters during the 5–18 weeks period could be one of the several reasons for decreased spontaneous behavioral CS during this period. The spontaneous electrographic NCS containing stage-2 spikes and the duration of such episodes observed in this study were similar to the EEG pattern described for the rat kainate model [71]. Also this pattern had the features of cortical EEG reported from the guinea pig kainate model [56], mouse kainate model [54], mouse model for absence seizures [72] and to some extent the EEG patterns corresponding to grade 2/3 seizures observed in the rat TBI model [73]. However, future studies of direct recording from the hippocampus, the entorhinal cortex and the amygdala are required to confirm the source of electrographic NCS and behavioral CS as demonstrated in other TLE models [10, 13, 31, 33, 56, 57].

In conclusion, the C57BL/6J mouse kainate model of epilepsy described in this study is useful to screen drugs for a short term course for behavioral CS. This model is also useful as a chronic complex seizure model to test long term effects of drugs on spontaneous electrographic NCS. Because of immediate epileptogenesis in the C57BL/6J mouse model, it provides an early window of opportunity for intervention and reduces the duration of experiments. In contrast to rat models, the mouse model also reduces the amount of drugs. Since some of the transgenic mice are also bred on C57BL/6J genetic background, this model is useful as a critical wild-type control for transgenic mice studies in epilepsy research.

## Supporting Information

Three videos (S1–3 Videos) demonstrate three types of spontaneous recurrent CS type-5 (wild running, rigidity, jumping, lateral recumbence). In all three videos, spontaneous CS can be observed at the last 30–55 seconds. The recordings prior to this reveal various spiking activities and their associated changes in the power spectrum.

S1 Video This video is an episode of a spontaneous stage- 5 seizure was observed on day 10 post-SE (the mouse marked MT6). The seizure can be observed between 30–48 seconds of the screen-captured video. The top panel (above the EEG trace) are the power bands and below the EEG trace are the activity counts/minute. During the CS, the power in the gamma band (green), the theta band (pink) and the delta band (in brown) increases that corresponds with the EEG spiking (high frequency and high amplitude), and the increased activity counts (light blue- at the bottom of the EEG trace). The high amplitude spikes corresponds to “popcorn”- type behavior followed by a typical postictal depression.
(MP4)

S2 Video An episode of a spontaneous behavioral CS type 5 on day 28 post-SE (the mouse marked MT8). The stage 5 seizure occurs between 30–53 seconds of the recording. After wild running and jumping (observe the increased amplitude on the EEG during the jump), the mouse becomes rigid which corresponds to the low amplitude EEG and a rapid drop in the activity counts.
(MP4)

S3 Video A spontaneous behavioral CS episode on day 20 (the mouse marked MT5). The stage 5 seizure occurs between 30–53 seconds of the recording.
(MP4)
